# Ferulic Acid Induces Th1 Responses by Modulating the Function of Dendritic Cells and Ameliorates Th2-Mediated Allergic Airway Inflammation in Mice

**DOI:** 10.1155/2015/678487

**Published:** 2015-10-01

**Authors:** Chen-Chen Lee, Ching-Chiung Wang, Huei-Mei Huang, Chu-Lun Lin, Sy-Jye Leu, Yueh-Lun Lee

**Affiliations:** ^1^Department of Microbiology and Immunology, School of Medicine, China Medical University, Taichung 40402, Taiwan; ^2^School of Pharmacy, College of Pharmacy, Taipei Medical University, Taipei 11031, Taiwan; ^3^Graduate Institute of Medical Sciences, College of Medicine, Taipei Medical University, Taipei 11031, Taiwan; ^4^Department of Microbiology and Immunology, College of Medicine, Taipei Medical University, Taipei 11031, Taiwan

## Abstract

This study investigated the immunomodulatory effects of ferulic acid (FA) on antigen-presenting dendritic cells (DCs) *in vitro* and its antiallergic effects against ovalbumin- (OVA-) induced Th2-mediated allergic asthma in mice. The activation of FA-treated bone marrow-derived DCs by lipopolysaccharide (LPS) stimulation induced a high level of interleukin- (IL-) 12 but reduced the expression levels of the proinflammatory cytokines IL-1*β*, IL-6, and tumor necrosis factor- (TNF-) *α*. Compared to control-treated DCs, FA significantly enhanced the expressions of Notch ligand Delta-like 4 (Dll4), MHC class II, and CD40 molecules by these DCs. Furthermore, these FA-treated DCs enhanced T-cell proliferation and Th1 cell polarization. In animal experiments, oral administration of FA reduced the levels of OVA-specific immunoglobulin E (IgE) and IgG_1_ and enhanced IgG_2a_ antibody production in serum. It also ameliorated airway hyperresponsiveness and attenuated eosinophilic pulmonary infiltration in dose-dependent manners. In addition, FA treatment inhibited the production of eotaxin, Th2 cytokines (IL-4, IL-5, and IL-13), and proinflammatory cytokines but promoted the Th1 cytokine interferon- (IFN-) *γ* production in bronchoalveolar lavage fluid (BALF) and the culture supernatant of spleen cells. These findings suggest that FA exhibits an antiallergic effect via restoring Th1/Th2 imbalance by modulating DCs function in an asthmatic mouse model.

## 1. Introduction

Asthma is a heterogeneous chronic inflammatory lung disease that is characterized by various airway obstructions, bronchial hyperresponsiveness, and airway inflammation. It is recognized that Th2 cells and their cytokines (interleukin- (IL-) 4, IL-5, and IL-13) are responsible for initiating and maintaining Th2-associated asthma [1]. These Th2 cytokines induce an inflammatory cascade that comprises allergen-specific immunoglobulin (Ig)E production, mast cell activation, eosinophil recruitment, and airway hyperresponsiveness (AHR) [2]. In addition to Th2-cell effects, dendritic cells (DCs), as professional antigen-presenting cells (APCs), play an important role in antigen presentation in the airways, and the expression of costimulatory molecules and cytokine profile by DCs can determine whether T cells differentiate into type 1 T-helper (Th1) cells, Th2 cells, or regulatory T cells (Tregs) [3, 4]. In addition, the ability of DCs to polarize Th2 responses may be enhanced by engagement of Notch receptors at the surface of T cells with ligands Jagged on DCs [5]. Therefore, inhibition of Th2 effector responses by modulating DCs maturation and function is considered a promising immunomodulatory strategy to treat Th2-associated allergic asthma.

Ferulic acid (trans-4-hydroxy-3-methoxycinnamic acid; FA; molecular weight 194.18) belongs to the family of phenolic acids and is widely found in vegetables, fruits, and some beverages such as coffee and beer [6]. Moreover, FA is also a component of Chinese medicinal herbs, such as* Angelica sinensis*,* Cimicifuga racemosa*, and* Ligusticum chuanxiong*. The daily amount of FA ingested was calculated to be around 150~250 mg [7]. After oral ingestion, FA is quickly absorbed and reaches a peak plasma concentration within 30 min [8]. Because of its antioxidative and anti-inflammatory activities, FA was shown to have therapeutic effects in various diseases such as Alzheimer's disease, cancer, diabetes mellitus, and arthritis [9–12]. Therefore, the abundant dietary sources, the relatively low toxicity, and potential bioactivities of FA may be capable of influencing immune cell functions in allergic immune responses and provide possible alternative options for relieving allergic asthma-associated symptoms. However, to date, the protective effects of FA against chronic inflammatory lung diseases, such as asthma, and the underlying mechanisms are still unavailable.

In the current study, we attempted to clarify whether treatment with FA can alter the phenotype and regulatory ability of DCs on T cells and achieve antiallergic effects to alleviate the development of airway symptoms in a mouse model of allergic asthma. Our data revealed that FA modulates DC function to promote interferon- (IFN-) *γ* production by activated T cells and convert T cells with Th1 activity in Th2-driven allergic diseases. These findings provide insights into how FA affects the Th2-biased immune response and provide guidance on the use of FA as an antiallergic adjuvant in treating Th2-mediated allergic asthma.

## 2. Materials and Methods

### 2.1. Mice

Female BALB/c and C57BL/6 mice were purchased from the National Laboratory Animal Center (Taipei, Taiwan) and maintained in the Animal Center of Taipei Medical University. Animals were housed in group cages (4-5 animals per cage) with free access to food and water. The environment was controlled on a 12 h dark-light cycle at a temperature of 23 ± 2°C. Animal care and handling protocols were evaluated and approved by the Animal Committee of the College of Medicine, Taipei Medical University (approval number LAC-98-0158).

### 2.2. Preparation of Bone Marrow-Derived DCs (BMDCs)

DCs were obtained by culturing BALB/c bone marrow cells in RPMI-1640 containing 5% fetal bovine serum (FBS), glutamine, penicillin/streptomycin, murine IL-4 (1000 U/mL), and GM-CSF (500 U/mL) for 6 days. Nonadherent cells were harvested and their purity was identified by flow cytometry, gated on CD11c^+^ cells. The FACS analysis showed that there were 70%~80% of DCs in this cell population.

### 2.3. Determination of Cytokine and Chemokine Levels

On day 6, 10^6^ cells/mL of BMDCs were stimulated with lipopolysaccharide (LPS; 1 *μ*g/mL) plus various concentrations of FA (20, 100, and 400 *μ*M) which was purchased from Sigma-Aldrich (St. Louis, MO, USA) or LPS alone for 24 h to achieve activation before use in the cytokine or phenotypical assays. In the experiments, untreated DCs were cultured with medium alone as the control. Concentrations of IL-1*β*, IL-6, IL-10, IL-12, and tumor necrosis factor- (TNF-) *α* in culture supernatants were evaluated by enzyme-linked immunosorbent assay (ELISA) kits (IL-1*β*, IL-6, IL-12, and TNF-*α* from eBioscience, San Diego, CA, USA; IL-10 from Duoset, R&D Systems, Minneapolis, MN, USA). The concentration of cytokines was measured by converting the OD values of the samples to pg/mL values from the standard curve.

The levels of eotaxin, IL-1*β*, IL-4, IL-5, IL-6, IL-10, IL-13, TNF-*α*, and IFN-*γ* in bronchoalveolar lavage fluid (BALF) and culture supernatants of splenocytes were also determined by commercial ELISA kits (eotaxin, IL-4, IL-5, IL-13, and IFN-*γ* from Duoset, R&D Systems).

### 2.4. Quantitative Real-Time Polymerase Chain Reaction

On day 6 of culture, BMDCs were collected and 10^6^ cells/mL were treated with LPS (1 *μ*g/mL) and various concentrations of FA (20, 100, and 400 *μ*M) or LPS alone for 6 h. Untreated DCs were cultured with medium alone as a control. After incubation, cells were collected, and total RNA was isolated according to the TRIzol method (Sigma-Aldrich, St. Louis, MO, USA). Complementary (c)DNA was synthesized from total RNA using the cDNA RT kit (Applied Biosystems, Foster City, CA, USA). Real-time PCR was performed with cDNA using the PCR master mix and TaqMan assays (Applied Biosystems). qPCR detection of mouse GAPDH, Delta1, Delta4, Jagged1, and Jagged2 was conducted in triplicate using an Applied Biosystems 7900 PCR system. Quantification was carried out using GAPDH gene expression as an internal normalization control, and the fold change in transcription was calculated using the comparative threshold cycle (C_T_) method, 2^−ΔΔC_T_^.

### 2.5. Flow Cytometric Analysis

Cytometric analysis was performed with a BD FACSort cell analyzer and CellQuest (BD Biosciences) software. DCs were stained with antibodies in phosphate-buffered saline (PBS) for 30 min at 4°C and washed with 2 mL of PBS. Then, cells were suspended in 0.5 mL of PBS with 0.1% sodium azide. Staining with isotype control antibodies was performed in all experiments. DCs were gated according to the standard forward- and side-scatter profiles of CD11c^+^ large cells. Anti-CD11c-fluorescein isothiocyanate (FITC), anti-CD40-FITC, anti-CD86-FITC, anti-CD11c-phycoerythrin (PE), anti-I-A/I-E (MHC class II)-PE, anti-CD80-PE, and anti-Jagged1-PE monoclonal antibodies (mAbs) were purchased from eBioscience; anti-Delta1-PE, anti-Delta4-PE, and anti-Jagged2-PE mAbs were purchased from BioLegend.

### 2.6. Mixed Lymphocyte Reaction (MLR)

This assay can be used to evaluate the stimulatory ability of DCs to alter T-cell proliferation and cytokine production. Approximately 10^6^ day-6 BMDCs derived from BALB/c mice were treated with LPS (1 *μ*g/mL), LPS plus FA (400 *μ*M), or medium only. After 24 h of culture, DCs were irradiated with 3000 rad (^137^Cs source). Allogeneic naïve CD4^+^ T cells from female C57BL/6 mice were purified by using CD4^+^ T-cell isolation kit (Miltenyi Biotec, Auburn, CA, USA). Then CD4^+^ T cells (2 × 10^5^ cells/well) were cultured with different proportions of irradiated DCs (0.5 × 10^4^, 10^4^, 2 × 10^4^, and 2 × 10^4^ cells/well) in 96-well round-bottom plates for 2 days. Tritiated thymidine (1 *μ*Ci/well, New England Nuclear, Boston, MA, USA) incorporation for 16 hours was determined with a dry scintillation counter (Packard Instrument, Meriden, CT, USA). Additionally, levels of IL-5 and IFN-*γ* production in the culture supernatant were assayed by ELISA kits.

### 2.7. Experimental Protocol for the Th2-Cell-Mediated Allergic Asthma Model

Female BALB/c mice at 6 weeks of age (weight range 19~20 g) were intraperitoneally (i.p.) sensitized with 50 *μ*g OVA (grade V; Sigma-Aldrich, St. Louis, MO) plus 2 mg of aluminum hydroxide (Thermo Scientific, Rockford, IL, USA) on day 1 and boosted with 25 *μ*g OVA in the same dosage of adjuvant on days 14 and 28. Subsequently, mice were intranasally (i.n.) challenged with 100 *μ*g OVA on days 43 and 44. Finally, mice were exposed to an OVA aerosol challenge on days 45, 46, and 47 by inhalation 5% OVA in a normal saline using an atomizer (DeVilbiss Health Care, Somerset, PA, USA) for 30 min, and AHR was measured 1 d after the final challenge (on day 48). To examine the effects of various dosages (25, 50, and 100 mg/kg of body weight) of FA, three groups of mice (FA (25), FA (50), and FA (100)) were, respectively, orally fed three doses of FA on days 21 to 47. OVA-sensitized and OVA-challenged mice were fed sterilized water instead of FA as a positive control (PC). Negative control (NC) mice were injected with PBS and challenged with OVA. During the experiment, no abnormal symptoms or toxic effects were observed in these groups. Serum samples were collected on days 1, 7, 28, 35, and 48.

### 2.8. Serum Antibody Assay

OVA-specific IgE, IgG_1_, and IgG_2a_ antibody titers in sera were determined by ELISA (Becton Dickinson Biosciences, San Jose, CA, USA) as described previously [13]. In brief, 96-well microtiter plates were coated with OVA (10 *μ*g/mL) in NaHCO_3_ buffer (pH 9.6) at 4°C overnight, and then treated with mouse sera followed by biotin-conjugated anti-mouse IgE, IgG_1_, or IgG_2a_ (PharMingen, San Diego, CA) for 1 h at 37°C. Then, avidin-horseradish peroxidase (1 : 5000, Pierce Biotechnology, Rockford, IL, USA) was added to each well for another 30 min at room temperature. Finally, the reaction was developed by 2,2′-azino-bis (3-ethylbenzthiazoline-6-sulfonic acid) (Sigma-Aldrich, St. Louis, MO) and the absorbance was determined at 405 nm. The levels of antibody were compared with OVA-specific IgE, IgG_1_ and IgG_2a_ standard serum with predetermined concentrations (immunoglobulin concentrations: IgE = 1.5 *μ*g/mL, IgG_1_ = 32.5 *μ*g/mL IgG_2a_ = 15.2 *μ*g/mL). The concentration of standard serum was arbitrarily assigned as 1 ELISA unit (1 EU).

### 2.9. Measurement of Airway Responsiveness

At 24 h after the final aerosol OVA exposure,* in vivo* lung function was measured by detecting changes in airway resistance in response to increasing doses of aerosolized methacholine (MCh, Sigma-Aldrich) in anesthetized mice as described previously [14]. MCh aerosol was generated with a nebulizer and administrated directly through the ventilator for 3 minutes. Airway reactivity was then monitored and data were expressed as the pulmonary resistance (*R*_*L*_) to estimate airway resistance.

### 2.10. Analysis of the Cellular Composition of BALF

After measuring the pulmonary function parameters, lungs of mice were lavaged three times with 1 mL of HBSS and the lavage fluids were centrifuged. The BALF supernatants were further assayed by the ELISA method, and cell pellets were resuspended in 1 mL HBSS. Total cells counts and differential counts of cytospin preparations stained by Liu's stain solution (Chi I Pao, Taipei, Taiwan) were determined by microscopy. At least 200 cells were counted and identified as macrophages, eosinophils, neutrophils, and lymphocytes under ×200 magnification. Because macrophages and lymphocytes have a similar staining profile, the different cell types were identified based on their morphology and size. The lymphocyte has a round nucleus and a high nuclear to cytoplasmic (N : C) ratio. Macrophages have a relatively low N : C ratio, and the cell size is larger (18–30 *μ*m in diameter) than lymphocytes (6 *μ*m in diameter).

### 2.11. Histological Examination of Lung Tissues

After being lavaged, the lungs were immediately inflated with 10% formalin, fixed for 24 hours and embedded in paraffin. For histological examination, 5 *μ*m-thick sections were cut, placed on glass slides, deparaffinized, and sequentially stained with hematoxylin-eosin (H&E) to evaluate inflammatory cell infiltration, or with periodic acid-Schiff (PAS) to identify goblet cells. Light microscopy was used for the histopathological assessment.

### 2.12. Statistical Analysis

Experimental results are presented as the mean ± standard error of the mean (SEM). Statistical analysis was performed using one-way analysis of variance (ANOVA) followed by Dunnett's post hoc test. A *p* value of less than 0.05 was considered to be significant.

## 3. Results

### 3.1. FA Treatment Enhanced Th1-Polarizing Cytokine Production and Reduced Proinflammatory Cytokine Secretion by LPS-Stimulated DCs

**Figure 1:**
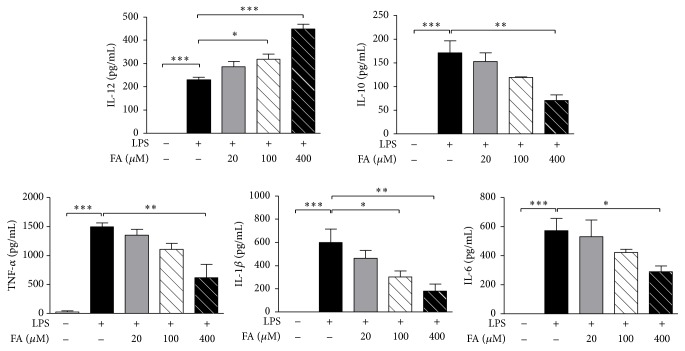
Ferulic acid modulates cytokine secretion in lipopolysaccharide-stimulated dendritic cells. On day 6 of culture, bone marrow-derived dendritic cells (BMDCs) were collected and treated with RPMI medium 1640, lipopolysaccharide (LPS, 1 *μ*g/mL) alone, or LPS plus various concentrations of FA (20, 100, and 400 *μ*M) for 24 h. Culture supernatants were collected, and cytokine profiles of DCs were measured by an ELISA. Results from three independent experiments are shown, and results are expressed as the mean ± SEM. ^*∗*^ *p* < 0.05, ^*∗∗*^ *p* < 0.01, and ^*∗∗∗*^ *p* < 0.001 versus LPS-treated DCs.

Before testing the effects of FA on BMDCs, we examined the effect of FA on cell viability. After 72 h of treatment with different doses of FA (0~5000 *μ*M) in BMDCs, cell numbers were counted and cell viability was determined by using a MTT assay. Results showed that the highest concentration (400 *μ*M) of FA used in the following experiments was not cytotoxic (Figure S1 in Supplementary Material available online at http://dx.doi.org/10.1155/2015/678487). We then addressed the impact of FA on the production of cytokines by DCs. LPS has been described as an inducer of DC activation and maturation. We used LPS as a positive control in this study. When BMDCs were treated with FA alone, there was no effect on cytokine production (unpublished data). Then, BMDCs were exposed to FA (20, 100, and 400 *μ*M) following LPS (1 *μ*g/mL) stimulation. In the presence of FA, dose-dependent increased levels of the Th1-polarizing cytokine IL-12 were observed in LPS-stimulated DCs (Figure 1). However, a dose-dependent decrease in IL-10 production was detected in DCs treated with FA plus LPS. We then examined the impact of FA on the production of proinflammatory cytokines, and as depicted in Figure 1, FA obviously inhibited TNF-*α*, IL-*β*, and IL-6 release by LPS-stimulated DCs.

### 3.2. FA Enhanced Notch Ligand Delta Expression by LPS-Stimulated DCs

**Figure 2:**
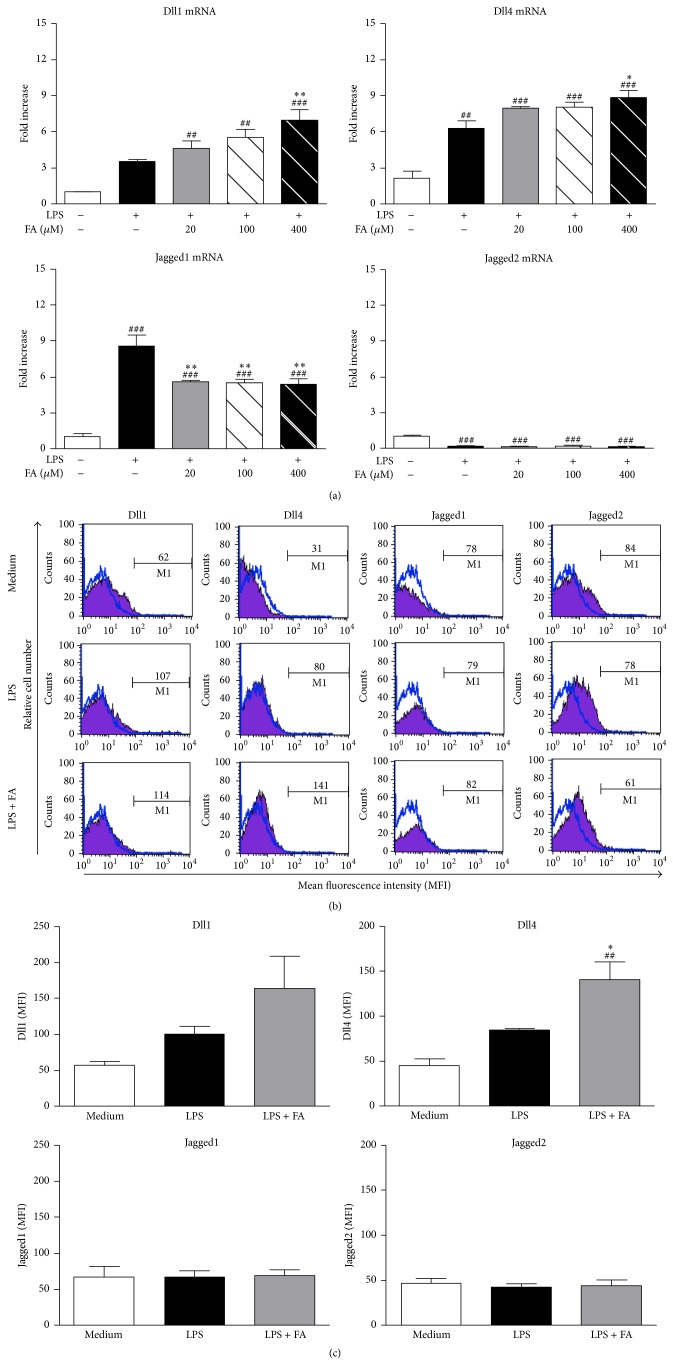
Effects of ferulic acid on expressions of Notch ligands in lipopolysaccharide-stimulated dendritic cells. (a) On day 6 of culture, BMDCs were left untreated or treated with LPS (1 *μ*g/mL) alone or LPS plus various concentrations of FA (20, 100, and 400 *μ*M). Untreated immature DCs served as the medium group. Cells were harvested after 5 h, and Jagged1, Jagged2, Dll1, and Dll4 mRNA accumulations were measured using a real-time RT-PCR. Results from three independent experiments are shown, and results are expressed as the mean ± SEM. ^##^ *p* < 0.01, ^###^ *p* < 0.001 versus medium-treated DCs. ^*∗*^ *p* < 0.05, ^*∗∗*^ *p* < 0.01, and ^*∗∗∗*^ *p* < 0.001 versus LPS-treated DCs. (b) Medium-treated DCs, LPS-treated DCs, and FA (400 *μ*M) plus LPS-treated DCs were cultured for 24 h. Then cells were harvested, and surface Notch ligand expressions were analyzed by flow cytometry. Each histogram shown is from one representative experiment performed. Values shown in the flow cytometric profiles are the mean fluorescence intensity (MFI) indexes. Cells were gated on CD11c, and the incidence of CD11c^+^ cells expressing the Notch ligands is indicated within each histogram. (c) The quantification of surface Notch ligand expressions is analyzed by flow cytometry. Results from three independent experiments are shown and are expressed as the mean ± SEM. ^##^ *p* < 0.01 versus medium-treated DCs. ^*∗*^ *p* < 0.05 versus LPS-treated DCs.

Variations in expressions of the Notch ligands, Delta (Delta-like 1 [Dll1] and Delta-like 4 [Dll4]) and Jagged families (Jagged1 and Jagged2), by mature DCs were shown to be crucial for T-cell differentiation. It was established that the Dll group directs T-cell polarization toward Th1, whereas the Jagged group promotes Th2 or Treg responses [15–18]. Thus, we investigated whether FA treatment cooperated with or interfered with their expressions, as assessed using a real-time RT-PCR. After LPS stimulation, DCs expressed higher levels of Dll1, Dll4, and Jagged1 mRNA synthesis compared to untreated DCs (Figure 2(a)). Additionally, compared to LPS-stimulated DCs, LPS combined with FA (400 *μ*M) treatment induced higher amounts of Dll1 and Dll4 transcripts in DCs. In contrast, exposure of DCs to LPS plus FA stimulation significantly reduced Jagged1 mRNA synthesis, although there was no interruption in Jagged2 transcripts. We further examined the surface expressions of these Notch ligands on DCs by performing FACS analyses. Compared to untreated DCs (medium group), a higher level of Dll1 was detected in LPS/FA-stimulated DCs (LPS+FA group), although there was no significant difference between the groups (Figures 2(b) and 2(c)). Notably, our data showed that the pattern of protein expression of Dll4 was significantly correlated with mRNA expression. Expressions of Jagged1 and Jagged2 showed no difference among the groups.

### 3.3. Effects of FA on the Maturation and Activation of LPS-Stimulated DCs

**Figure 3:**
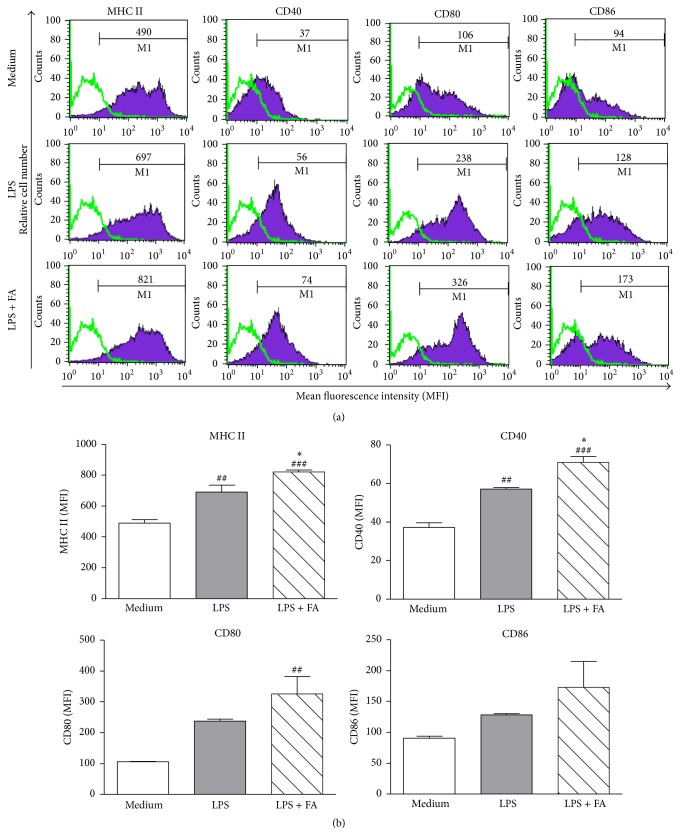
Ferulic acid treatment affects the maturation phenotype of lipopolysaccharide-stimulated dendritic cells. DCs were treated with medium, LPS (1 *μ*g/mL) alone or LPS plus FA (400 *μ*M) for 24 h. After incubation, cells were collected, and expressions of MHC class II, CD40, CD80, and CD86 by DCs were analyzed using flow cytometry. (a) Values shown in the flow cytometric profiles are the mean fluorescence intensity (MFI). Cells were gated on CD11c, and the incidence of CD11c^+^ cells expressing the surface marker is indicated within each histogram. Values shown are from one representative experiment of three independent experiments performed. (b) The MFI was calculated, and results are expressed as the mean ± SEM from three independent experiments. ^##^ *p* < 0.01, ^###^ *p* < 0.001 versus medium-treated DCs. ^*∗*^ *p* < 0.05, ^*∗∗∗*^ *p* < 0.001 versus LPS-treated DCs.

To investigate whether FA modulated the maturation and activation of mouse BMDCs* in vitro*, we compared the phenotype of mouse DCs treated with medium, LPS alone, or LPS plus FA (400 *μ*M) for 24 h. As shown in Figures 3(a) and 3(b), expressions of MHC class II and CD40 molecules during maturation were markedly upregulated in LPS/FA-treated DCs compared to LPS-treated DCs or untreated DCs, respectively (Figure 3 **)**. Furthermore, expressions of CD80 and CD86 molecules in LPS/FA-treated DCs were higher than those of untreated DCs, although the difference was not significant.

### 3.4. FA- and LPS-Treated DCs Enhanced T-Cell Proliferation and Th1 Polarization

**Figure 4:**
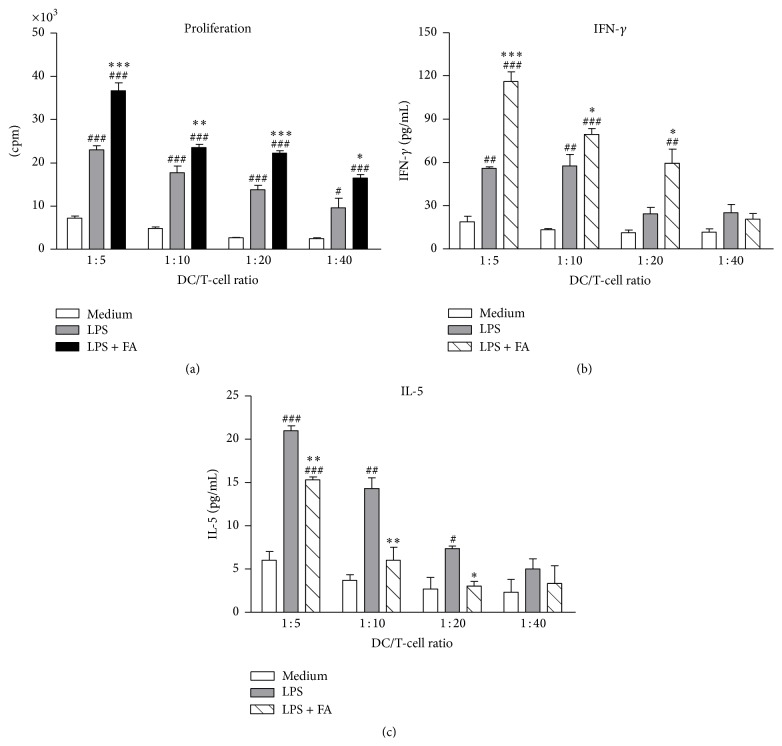
Ferulic acid-modulated dendritic cells enhanced T-cell proliferation and Th1 cell polarization. (a) Modulatory effects of FA on T-cell proliferation. DCs were treated with medium, LPS (1 *μ*g/mL), or LPS plus FA (400 *μ*M) for 24 h. Allogeneic naïve CD4^+^ T cells (2 × 10^5^ cells/well) were cocultured with different proportions of *γ*-irradiated immature DCs, LPS-pulsed DCs, or LPS plus FA-treated DCs at DC/T-cell ratios of 1 : 5, 1 : 10, 1 : 20, and 1 : 40 in 96-well round-bottomed plates. After 2 days of culture, cells were pulsed with 1 *μ*Ci/well of [^3^H]-thymidine for 16~18 h. Specific incorporation of [^3^H]-thymidine was determined with a *β*-counter, and results are expressed as counts per minute (cpm). (b) Modulatory effects of FA on T-cell cytokine secretion. Concentrations of IFN-*γ* and IL-5 secreted by naïve CD4^+^ T cells, which were cocultured with *γ*-irradiated DCs in 96-well plates for 2 days, were determined by an ELISA. Results in all panels are expressed as the mean ± SEM of three independent experiments. ^#^ *p* < 0.05, ^##^ *p* < 0.01, and ^###^ *p* < 0.001 versus medium-treated DCs. ^*∗*^ *p* < 0.05, ^*∗∗*^ *p* < 0.01, and ^*∗∗∗*^ *p* < 0.001 versus LPS-treated DCs.

Mature DCs have the capacity to induce proliferation of activated T cells at much higher levels than immature DCs. To determine the ability of DCs to induce T-cell proliferation and cytokine production, an allogeneic MLR assay was used. DCs from BALB/c mice were treated with medium, LPS alone, or LPS plus FA and then cocultured with C57BL/6 mouse-derived naïve CD4^+^ T cells at various ratios (DC/T-cell ratios of 1 : 5, 1 : 10, 1 : 20, and 1 : 40)* in vitro*. As shown in Figure 4(a), treatment with FA of DCs cultured in the presence of LPS stimulated T-cell proliferative responses more effectively than those of LPS-treated DCs (Figure 4(a)). Additionally, we found that FA modulated the secretion of IFN-*γ* and IL-5 by activated CD4^+^ T cells. FA- and LPS-treated DCs markedly enhanced T-cell secretion of IFN-*γ* in the supernatant compared to LPS-treated DCs (Figure 4(b)). In contrast, FA plus LPS-treated DCs significantly suppressed IL-5 release by T cells in a dose-dependent manner. These results showed that FA can enhance the Th1 response through DCs.

We also investigated whether FA could directly affect the cytokines production by T cells. At first, we evaluated the possible cytotoxic effect of FA on CD4^+^ T cells. After treatment with 1 to 50 *μ*M of FA for 72 h, T cells did not show any change on cell viability as detected by MTT assay (Figure S2A). To analyze the direct effect of FA on T cells, CD4^+^ T cells were treated with FA (5, 10, and 50 *μ*M) and activated by anti-CD3/anti-CD28 antibodies. Compared to control T cells (anti-CD3/anti-CD28 treatment group), activated T cells cultured in the presence of FA did not show any apparent differences in cytokine production (IFN-*γ*, IL-10, and IL-5) (Figure S2B). These results indicated that FA could not directly inhibit or promote cytokine production from T cells.

### 3.5. FA Reduced Serum Anti-OVA IgE and IgG_1_ and Enhanced Anti-OVA IgG_2a_ Levels in an Animal Model of Asthma

**Figure 5:**
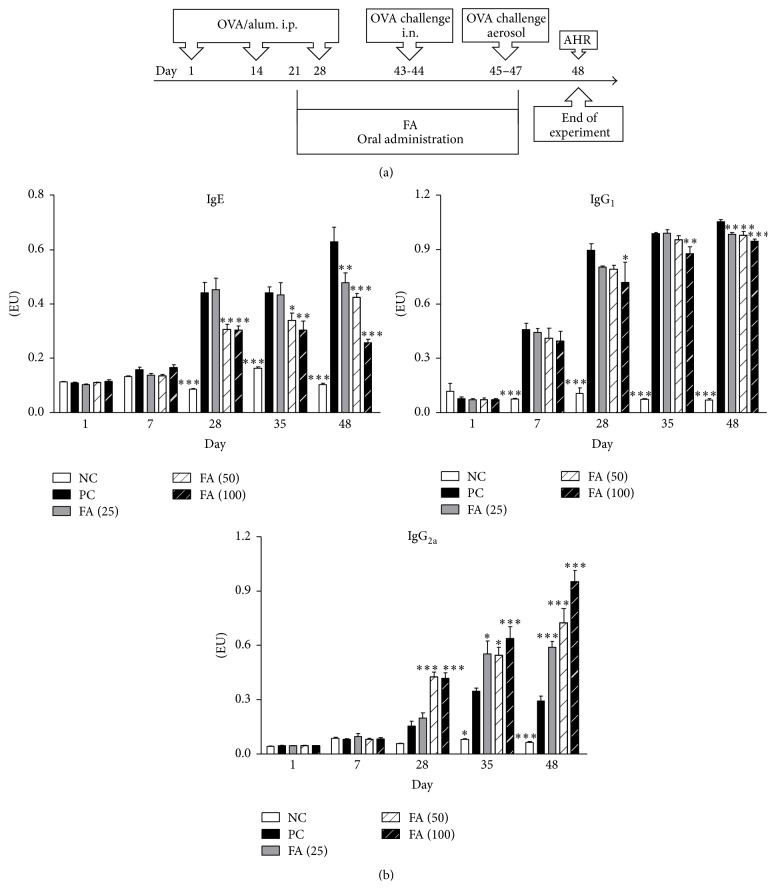
Anti-OVA antibody expression levels of ferulic acid-treated OVA-induced allergic asthmatic mice. (a) Brief description of the protocol of animal sensitization and challenge. On days 1, 14, and 28, all groups of mice were sensitized by an intraperitoneal injection of the OVA allergen. Three groups of mice (FA (25), FA (50), and FA (100)) were, respectively, orally fed 25, 50, and 100 mg/kg of FA on days 21 to 47. Positive control (PC) mice were administered sterilized water instead of FA. Then, mice were intranasally challenged with OVA on days 43 and 44. Subsequently, mice were exposed to OVA aerosols for 3 consecutive days, and AHR was measured 1 day after the last challenge. BALF was collected after measuring the AHR. Negative control (NC) mice were neither sensitized with OVA nor administered FA treatment but were challenged with OVA. (b) IgE, IgG_1_, and IgG_2a_ anti-OVA antibody expressions of all groups of mice were measured by an ELISA. Results are expressed as the mean ± SEM (*n* = 8 in each group). ^*∗*^ *p* < 0.05, ^*∗∗*^ *p* < 0.01, and ^*∗∗∗*^ *p* < 0.001 versus the PC group.

To investigate the* in vivo* impacts of FA on regulating immune responses of allergic diseases, groups of mice were exposed to OVA sensitization and challenge (Figure 5(a)). These OVA-sensitized mice were orally fed different doses (25, 50, and 100 mg/kg of body weight (BW)) of FA (FA (25), FA (50), and FA (100), resp.). PC mice received distilled water instead of FA. NC mice were neither sensitized with OVA nor administered FA treatment, although they received an OVA challenge. Serum samples were collected on indicated days, and OVA-specific IgE, IgG_1_, and IgG_2a_ serum antibody titers were determined by an ELISA. In mice, the Th2 cytokine, IL-4, promotes IgG_1_ and IgE production. However, the Th1 cytokine, IFN-*γ*, induces the production of IgG_2a_. Therefore, expression levels of subclasses (IgG_1_ and IgG_2a_) or classes (IgE) of antibodies can be used as markers to identify what type of Th response was induced in these mice. In the PC group, strong IgE and IgG_1_ production and low IgG_2a_ expression were induced (Figure 5(b)). In contrast, FA treatment efficiently inhibited OVA-specific IgE and IgG_1_ production. Notably, the administration of FA markedly upregulated IgG_2a_ synthesis compared to the PC group.

### 3.6. FA Decreased the Severity of AHR and Airway Inflammation

**Figure 6:**
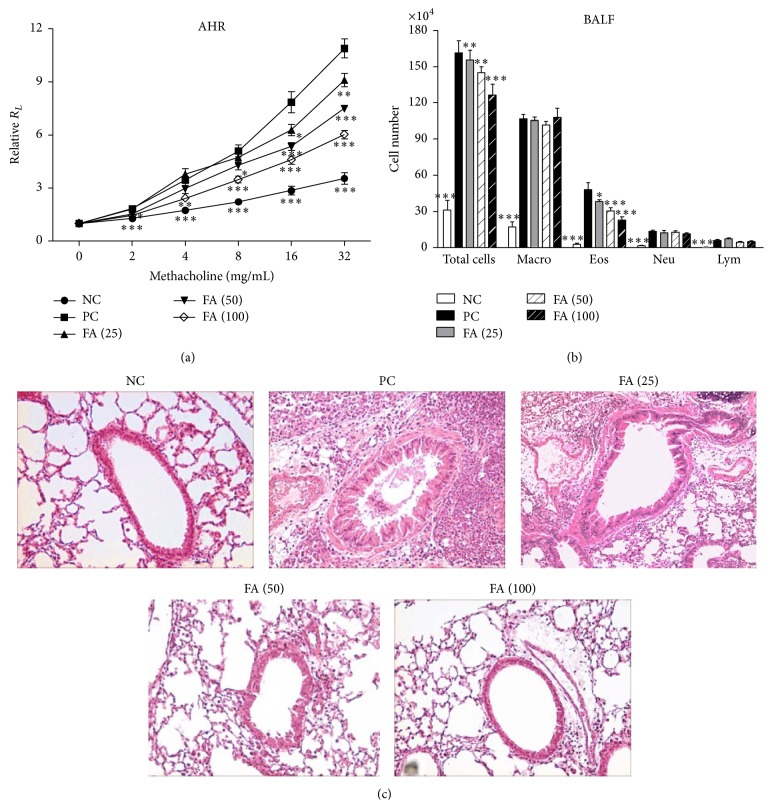
Effects of ferulic acid on the airway hyperresponsiveness and airway inflammation. (a) Oral administration of FA suppressed the development of AHR in OVA-sensitized mice. One day after the last OVA challenge, airway resistance was measured in response to increasing concentrations of methacholine (0~32 mg/mL) by invasive body plethysmography. Results are expressed as the mean ± SEM (*n* = 8 in each group) of the pulmonary resistance (*R*_*L*_) after PBS nebulization. (b) Changes in the cellular composition of the BALF of mice exposed to an aerosolized allergen. One day after measuring the pulmonary function parameters, each group of mice was sacrificed, and the BALF was collected. Cells were counted and classified as macrophages (Macro), eosinophils (Eos), neutrophils (Neu), and lymphocytes (Lym). Results are expressed as the mean ± SEM (*n* = 8 in each group). (c) Effect of FA on lung inflammatory cell infiltration in OVA-induced allergic asthmatic mice (H&E staining). Lung sections were obtained from NC mice, sterilized water-treated PC mice, and those treated with different doses of FA. Sections were stained with H&E for the morphological analysis. Tissues were examined by light microscopy (original magnification: ×200). ^*∗*^ *p* < 0.05, ^*∗∗*^ *p* < 0.01, and ^*∗∗∗*^ *p* < 0.001 versus the PC group.

To assess the antiallergic effects of FA on allergic asthma, both the AHR and accumulation of inflammatory cells in BALF were investigated. One day after the last OVA challenge, the airway responsiveness to aerosolized MCh of each group of mice was measured. PC mice, which were administered distilled water instead of FA and sensitized and challenged with OVA, developed markedly increased airway responsiveness to MCh inhalation compared to that of nonallergic NC mice (Figure 6(a)). However, oral treatment with FA dose dependently alleviated the development of AHR compared to the PC group. Furthermore, in the NC group, cells in the BALF were mostly composed of macrophages (Figure 6(b)). On the other hand, in the PC group, exposure to aerosolized OVA-induced marked increases in cell numbers of eosinophils in the BALF. In contrast, mice administered FA showed significantly reduced increases in eosinophils. Furthermore, a pathological examination of lung tissue sections from the PC group exhibited increased numbers of inflammatory cells around the peribronchiolar region (Figure 6(c)), relative to the NC group. Of note, lungs of mice administered FA demonstrated efficient inhibitory effects on peribronchial inflammation.

### 3.7. The Influence of FA on OVA-Induced Chemokine and Cytokine Secretions in BALF

**Figure 7:**
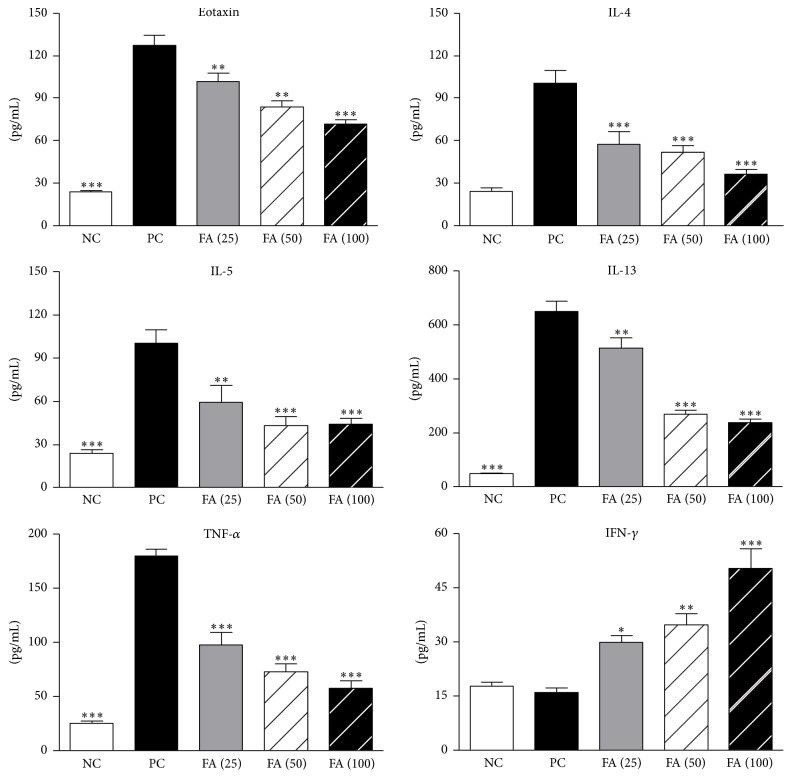
Effect of ferulic acid on levels of chemokines and cytokines in the bronchoalveolar lavage fluid. Immediately after measuring the pulmonary function parameters, each group of mice was sacrificed, and BALF was collected and analyzed for eotaxin, IL-4, IL-5, IL-13, IFN-*γ*, and TNF-*α* contents by an ELISA. Results are expressed as the mean ± SEM (*n* = 8 in each group). ^*∗*^ *p* < 0.05, ^*∗∗*^ *p* < 0.01, and ^*∗∗∗*^ *p* < 0.001 versus the PC group.

Eotaxin is the most potent chemokine for recruiting eosinophils. Evaluation of the eotaxin level in BALF showed that administration of FA significantly reduced eotaxin secretion in a dose-dependent manner (Figure 7). This result indicated that FA suppressed eotaxin secretion and subsequently decreased numbers of recruited eosinophils. Furthermore, to determine the possible effect of FA on T-cell responses, we assessed levels of the important Th2 cytokines, IL-4, IL-5, and IL-13, in BALF. The data showed that FA treatment had dose-dependent suppressive effects on the production of Th2 cytokines compared to the PC group. Similarly, the expression level of TNF-*α* in the BALF also decreased in FA-treated mice. In contrast, we observed significant enhancement of BALF IFN-*γ* expression in mice treated with FA compared to the PC group.

### 3.8. Effects of FA on OVA-Induced Cytokine Secretions by Splenocytes

**Figure 8:**
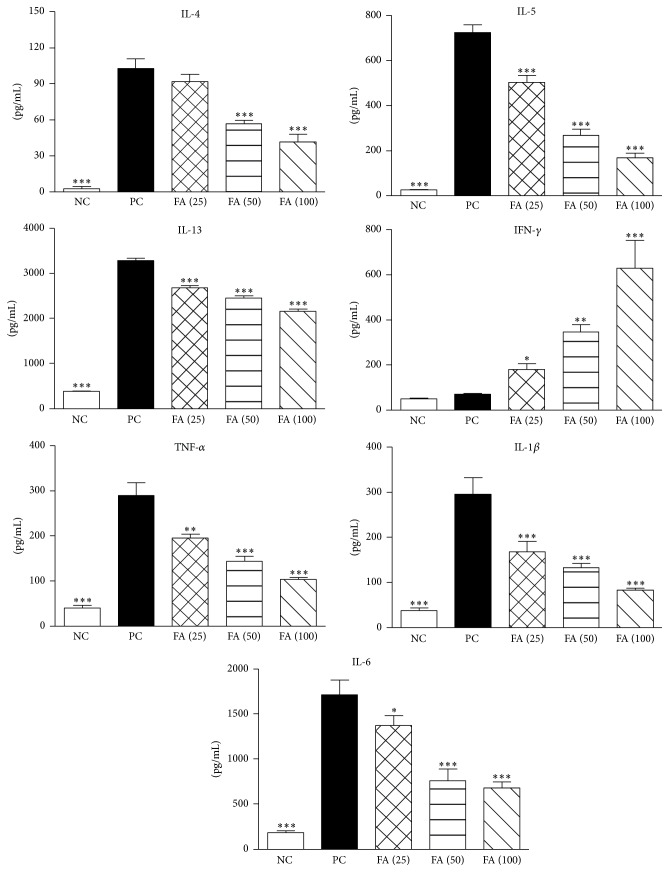
Effect of ferulic acid on the cytokine profile of OVA-restimulated splenocytes. Splenocytes (10^7^ cells/mL) from FA-treated and control groups were stimulated with 50 *μ*g/mL OVA in 24-well plates, and culture supernatants were collected after 72 h. Levels of cytokine production of IL-4, IL-5, IL-13, IFN-*γ*, TNF-*α*, IL-1*β*, and IL-6 were analyzed by an ELISA. Results are expressed as the mean ± SEM (*n* = 8 in each group). ^*∗*^ *p* < 0.05, ^*∗∗*^ *p* < 0.01, and ^*∗∗∗*^ *p* < 0.001 versus the PC group.

By studying the production of Th1 and Th2 cytokines in cell culture supernatants from the spleen, we determined whether it was possible to affect Th2 immune responses through inducing IFN-*γ*-producing T cells. Freshly isolated spleen cells from different groups of mice were cultured with OVA. After 3 days of culture, cell supernatants were collected and analyzed by an ELISA. Results showed that FA administration markedly inhibited IL-4, IL-5, and IL-13 production by splenocytes (Figure 8). Furthermore, the data demonstrated that OVA-sensitized mice treated with FA exhibited an obviously enhanced level of IFN-*γ* production. We also checked expressions of the proinflammatory cytokines, TNF-*α*, IL-1*β*, and IL-6, by splenocytes and found that three dosages of FA treatment significantly reduced production of these cytokines relative to mice in the PC group. Taken together, the above findings suggest that FA treatment might induce protective immunity against established Th2-mediated allergic asthma through induction of IFN-*γ* production by OVA-specific T cells.

## 4. Discussion

Asthma affects over 300 million people worldwide and constitutes heavy medical, social, and economic burdens, because its prevalence is continually increasing. Still, in many cases, although corticosteroid and *β*-agonists can improve asthma symptoms, their long-term use has adverse effects. Therefore, it is imperative to find substances, preferably nonsteroidal in nature and especially those derived from plants used as folk medicines or foods, which have therapeutic effects on allergic asthma and can be taken for a long time with no side effects.

FA is one of the most abundant phenolic acids in plants. It acts as a potent antioxidant which effectively scavenges free radicals and inhibits lipid peroxidation [19]. FA reduced hydrogen peroxide-induced lipid peroxidation in peripheral blood mononuclear cells, and this effect was more evident than that of other polyphenols such as caffeic acid and ellagic acid [20]. FA also increases the activity of enzymes that can counteract free radical-induced damage. Based on this evidence, FA was shown to have a protective role in many disorders, including cardiovascular dysfunctions, cancer, diabetes, and neurodegenerative diseases [21–23]. On the other hand, FA exerts anti-inflammatory properties by affecting the immune system. Hirabayashi et al. found that FA reduced IL-8 production in the influenza virus-infected RAW264.7 macrophage cell line [24]. In the presence of FA, respiratory syncytial virus- (RSV-) infected RAW264.7 cells showed reduced macrophage inflammatory protein- (MIP-) 2 production in a dose-dependent manner [25]. Further, FA inhibited the release of TNF-*α* from phytohemagglutinin-stimulated splenocytes [26]. These data indicate that FA inhibits inflammation by interfering with immune cells.

In the immune system, DCs are the most potent APCs and are critically involved in initiating primary immune responses and inducing T-cell responses. Thus, elucidating the immunomodulatory effect of FA on DCs could be crucial for understanding the impacts of FA on immune responses. As mature APCs, fully activated mature DCs display high levels of peptide-MHC class II complexes on their surfaces and upregulate surface levels of costimulatory molecules (CD40, CD80, and CD86). These mature DCs are activated through CD40 and produce high levels of IL-12 and TNF-*α* which drive Th1 cell differentiation [27]. In contrast, differentiation of Th2 cells depends on a low-IL-12 and high-IL-4 environment. IL-4 is not produced by DCs but by other cells, such as mast cells [28]. Similarly, differentiation of Th17 cells is also determined by the cytokine milieu. IL-1*β*, IL-6, and TGF-*β* program Th17 cell differentiation [29]. In our study, a FACS analysis of DCs cultured in the presence of FA plus LPS revealed upregulation of MHC class II and CD40 molecules. Further, these FA- and LPS-treated DCs exhibited increased production of IL-12. However, they did not release elevated levels of proinflammatory cytokines, such as TNF-*α*, IL-1*β*, or IL-6. Thus, from the above results, we predict that Th1 differentiation may preferably be driven by FA- and LPS-treated DCs. Although it is interesting to discuss which intracellular signaling pathway was involved in modulating gene expression of those cytokines in DCs by FA, further evidence is not available. MAP kinases (MAPKs) consist of three major groups, p38 MAPK, the c-Jun N-terminal kinase 1/2 (JNK1/2), and extracellular signal-regulated kinase 1/2 (ERK1/2), and previous reports indicate that these proteins play a role in regulating cytokine production from APCs [30]. One study reported that LPS instructs DCs to produce IL-12, which depends on the phosphorylation of p38 MAPK and JNK1/2 [31]. Thus, we predict that FA might selectively enhance IL-12 production via amplification of p38 MAPK or JNK1/2 pathway. In addition, FA can act as an antioxidant to reduce proinflammatory cytokine production by scavenging reactive oxygen species (ROSs) [21]. Thus, the inhibition of TNF-*α*, IL-1*β*, and IL-6 by FA might be mediated via a mechanism involving the blockage of ROS pathway. In contrast, it is possible that the upregulation of IL-12 production in DCs is through the inhibition of ROS activity. This explanation is supported by one report that H_2_O_2_, a major component of ROSs, downregulates IL-12 production in murine macrophages in response to stimulation with LPS plus IFN-*γ* [32]. Further study would be required to clarify this question.

In the current study, we found that FA treatment induced a higher expression level of Dll4 by DCs than those of other Notch ligands. In addition, coculture of CD4^+^ T cells with LPS-pulsed Dll4-stimulated DCs enhanced the differentiation of Th1 cells characterized by the secretion of increased levels of IFN-*γ*. Unstimulated DCs express low levels of Notch ligands. Under different conditions, the expression of Notch ligands can be induced towards preferential expression of either Jagged or Delta in response to different stimuli. Amsen et al. demonstrated that expression of Dll4 by DCs was correlated with the ability of LPS to polarize Th1 cells, whereas Jagged1 expression was correlated with the ability of LPS to polarize Th2 cells [5]. The discovery of the contribution of Notch signaling in Th1/Th2 differentiation opens a new pathway for regulating helper T-cell differentiation. Furthermore, Sun et al. showed that ectopic expression of Dll4 by BMDCs promoted Th1-cell differentiation and allowed them to strongly inhibit Th2 development [33]. In addition, inhibition of Th2-type cytokine IL-4 production allows Th cells activated by Dll4 to develop into Th1 cells through a pathway that is IL-12-independent. In summary, our results showed that stimulation via FA can modify the maturation profile and function of DCs, and high expression of Dll4 by these DCs enhanced their ability to activate naïve Th cells and promote Th1 cell development.

In the present study, for the first time, we explored the immunomodulatory effects of FA in a murine asthma model. Our study demonstrated that oral administration of 25 to 50 mg/kg of FA could get significant protective effects against Th2-cell-mediated allergic asthma in mice. FA is of low toxicity with LD_50_ of 2445 mg/kg and 2113 mg/kg body weight in male and female rats, respectively [34]. Although humans may consume as much as 80 to 165 mg FA per meal, low bioavailability after oral administration may limit the clinical use of FA. Another limitation is that many studies on humans were carried out by using foods containing FA rather than the purified FA. Thus, unfortunately, we could not compare with these studies to evaluate the suitable dose of FA applied in humans.

Our results clearly demonstrated that orally administered FA has an antiallergic effect via suppressing OVA-specific IgE production. Serum IgE is one of the most important clinical markers for allergic responses. Such a reduction in serum OVA-specific IgE would likely relieve the allergic syndrome mediated by mast cells [35]. Further, our present results showed that FA treatment significantly induced a reduction in the IL-4 level in mice. IL-4 is a switching factor for IgE production, and it also induces the rolling and adhesion of circulating eosinophils to endothelial cells [36]. Therefore, we speculated that FA may have an antiallergic effect on allergic asthma by suppressing IL-4 secretion which consequently reduces IgE production and eosinophil infiltration into the lungs.

Airway eosinophilic inflammation is one of the characteristics of bronchial allergic asthma. It is believed that eosinophil transmigration into the airways is orchestrated by cytokines, such as IL-4, IL-5, TNF-*α*, and IL-13, and is coordinated by specific chemokines, such as eotaxin [37, 38]. Specifically, IL-5 is the most important mediator that regulates eosinophilic inflammation through its effects on the proliferation, differentiation, and activation of eosinophils and as a signal for mobilization of eosinophils from the bone marrow into the lungs after an allergen challenge [39, 40]. Additionally, TNF-*α* and IL-1*β* are particularly important inflammatory mediators because they play major roles in coordinating mechanisms that command proinflammatory responses. Previous studies reported that levels of TNF-*α*, IL-1*β*, and IL-6 released in the BALF of asthmatic patients increased [41]. TNF-*α* and IL-1*β* can induce the synthesis of eotaxin in human lung epithelial cells [42]. The results of our study clearly showed that FA attenuated the overproduction of Th2-associated cytokines and downregulated eotaxin expression. Likewise, levels of these proinflammatory cytokines were markedly reduced in FA-treated asthmatic mice. As expected, consistent with results of the histological analysis of lung tissues, FA successfully alleviated lung inflammation and reduced infiltration of total inflammatory cells in peribronchiolar regions. Based on these findings, we speculated that FA inhibits the recruitment of inflammatory cells by reducing levels of Th2-type cytokines, chemokines, and proinflammatory cytokines, providing a possible mechanistic explanation for the observed profound regulatory effects of FA on the development of airway inflammation in this OVA-induced asthma model.

AHR is a major characteristic feature of asthma and a pathophysiologic consequence of the effect of inflammatory processes. In particular, IL-13 can directly cause AHR by affecting airway epithelial cells and airway smooth muscle cells [43, 44]. Herein, we found that BALF and spleen IL-13 levels decreased after oral administration of FA; therefore, it caused attenuation of AHR sensitized by an allergen in mice. IL-10 was shown to be produced by specialized subsets of Treg cells and the regulatory activities of T cells were found to be closely related to their IL-10-producing ability [45, 46]. In this murine asthma model, we detected the expression level of IL-10 in splenocytes. The data showed that FA treatment slightly reduced IL-10 production in splenocytes (Figure S3A). In addition, treatment with FA enhanced but not reduced the OVA-specific T-cell proliferative response (Figure S3B). These results implied that FA treatment did not induce Treg-cell response in this asthmatic animal model.

By administering FA, we demonstrated that FA-treated DCs stimulated T-cell proliferation and enhanced the generation of Th1 cells in an allogeneic MLR. In an animal study, we also showed that treatment of asthmatic mice with FA had inductive effects on Th1 responses, including anti-OVA IgG_2a_ and IFN-*γ* production. IFN-*γ*, which is secreted by Th1 cells, promotes the proliferation of Th1 cells and induces IgG_2a_ production by B cells. It inhibits Th2-cell proliferation* in vitro*. Many studies demonstrated that the exogenous delivery of IFN-*γ* suppresses recruitment of eosinophils and inhibits AHR and bronchial mucus production in asthmatic mice [47–49]. These data support the ability of IFN-*γ* to block Th2-cell cytokine production and CD4 T-cell recruitment to the lungs. To examine the precise mechanism of suppression, Nakagome et al. delivered IFN-*γ* to asthmatic mice using an IFN-*γ*-producing plasmid and reported that IFN-*γ* attenuated the OVA-induced Th2 immune response by suppressing DC functions, such as antigen presentation, cytokine production, and migration to the lungs [49]. Mitchell et al. used IFN-*γ* receptor-expressing transgenic mice and showed that IFN-*γ* acting through the airway epithelium blocked Th2-mediated mucus and inhibited eosinophil generation in the bone marrow [50]. IFN-*γ* is also considered to play some role in host defense against rhinovirus infection in asthmatic patients. It was speculated that a decrease in its production by peripheral blood mononuclear cells or by lung cells may result in an exacerbation of asthma [51, 52]. These findings suggest that IFN-*γ* may be beneficial in treating asthma by inhibiting virus-induced exacerbation. Although those studies indicated a novel role for IFN-*γ* as a broad immune suppressor to modulate immune responses in allergic inflammation, understanding the various mechanisms of IFN-*γ* regulation in Th2-mediated allergic asthma may define a pathway for new asthma therapies to target in the future.

## 5. Conclusions

In conclusion, the current study demonstrated the ability of FA to induce maturation of a distinct subset of DCs after* in vitro* activation with LPS. In addition, these DCs induced differentiation of IFN-*γ*-secreting Th1 cells* in vitro*. Furthermore, oral administration of OVA-induced asthma in recipient mice with FA that could lead to differentiation of a specific Th1 population able to downregulate reactivity mediated by IFN-*γ* production. Therefore, we concluded that the antiallergic effects of FA were due to the modulation of DCs, which promoted the differentiation of naïve T cells into Th1 cells thereby restoring the Th1/Th2 imbalance in a Th2-mediated allergic asthmatic mouse model. Thus, FA might become a very attractive immunomodulatory material that can supplement the human diet. However, further research is necessary to focus on the activation and inhibition mechanisms of FA to clarify its immunomodulatory activities on DCs.

## Supplementary Material

DCs were treated with 5 to 5000 uM FA for 72 h and cell viability was detected by MTT assay. Because FA showed no cytotoxicity at concentrations up to 500 uM in DCs, we used up to 400 uM FA for the rest of the experiments.
