# Corrigendum to “Ferulic Acid Induces Th1 Responses by Modulating the Function of Dendritic Cells and Ameliorates Th2-Mediated Allergic Airway Inflammation in Mice”

**DOI:** 10.1155/2017/1039829

**Published:** 2017-09-20

**Authors:** Chen-Chen Lee, Ching-Chiung Wang, Huei-Mei Huang, Chu-Lun Lin, Sy-Jye Leu, Yueh-Lun Lee

**Affiliations:** ^1^Department of Microbiology and Immunology, School of Medicine, China Medical University, Taichung 40402, Taiwan; ^2^School of Pharmacy, College of Pharmacy, Taipei Medical University, Taipei 11031, Taiwan; ^3^Graduate Institute of Medical Sciences, College of Medicine, Taipei Medical University, Taipei 11031, Taiwan; ^4^Department of Microbiology and Immunology, School of Medicine, College of Medicine, Taipei Medical University, Taipei 11031, Taiwan

In the article titled “Ferulic Acid Induces Th1 Responses by Modulating the Function of Dendritic Cells and Ameliorates Th2-Mediated Allergic Airway Inflammation in Mice” [1], affiliation number four was given incorrectly. The corrected affiliation is shown above.
