# Supplementary material for: Ferulic Acid Induces Th1 Responses by Modulating the Function of Dendritic Cells and Ameliorates Th2-Mediated Allergic Airway Inflammation in Mice
Source: Evid Based Complement Alternat Med. 2015 Oct 1;2015:678487. doi: 10.1155/2015/678487 (PMC4606409; doi:10.1155/2015/678487)
Supplement: Supplementary file 1 — DCs were treated with 5 to 5000 uM FA for 72 h and cell viability was detected by MTT assay. Because FA showed no cytotoxicity at concentrations up to 500 uM in DCs, we used up to 400 uM FA for the rest of the experiments. [file 678487.f1.pdf]

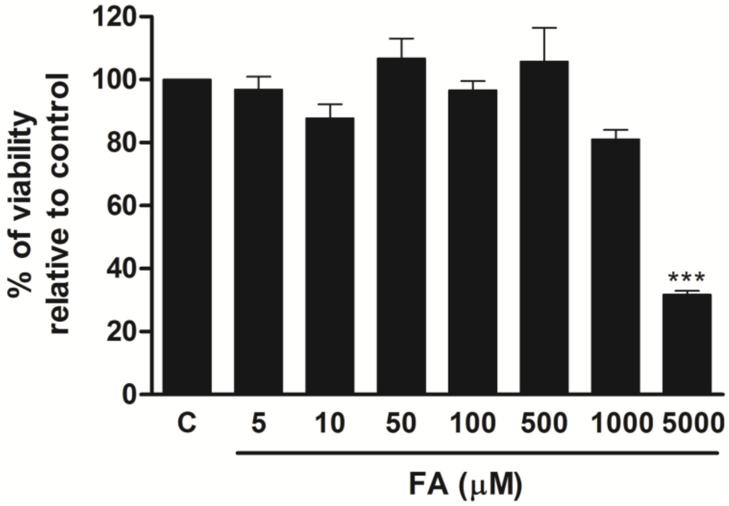

Figure S1: Cytotoxic effect of ferulic acid on dendritic cells. On day 6 of culture, bone marrow-derived dendritic cells (DCs) were treated with various concentrations of FA for 72 h. Untreated DCs were pulsed with medium alone as the control. These cells were incubated with 10 μl of 5 mg/mL of MTT for another 4 h, and then solubilized in DMSO. The amount of reduction was measured using a microplate reader at 570 nm. Results from three independent experiments are shown and are expressed as the mean ± SEM. *** $p < 0.001$ vs. control DCs.

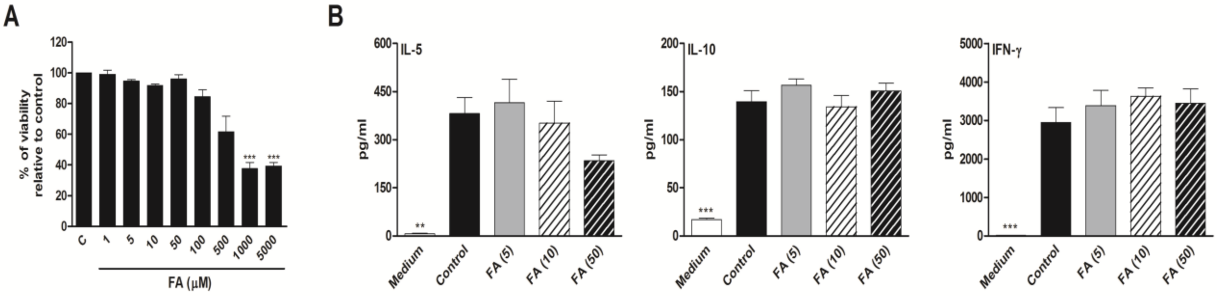

Figure S2: The direct effect of ferulic acid on CD4$^+$ T cells. (A) Cytotoxicity effect of FA on T cells. T cells were treated with different doses of FA for 72 h. Cell viability was detected by using MTT assay. Untreated T cells were as the control. Results from three independent experiments are shown and are expressed as the mean ± SEM. *** $p < 0.001$ vs. control T cells. (B) The direct effect of FA on T-cell cytokine production. T cells (1×10^6 cells/well) were cultured in 24-well plates and stimulated with anti-CD3 (1 μg/ml)/anti-CD28 (1 μg/ml) antibodies in the presence of FA (5, 10, and 50 μM) for another 72 h. T cells stimulated with anti-CD3/anti-CD28 alone as the control. The culture supernatants were collected and analyzed by an ELISA. Results from triplicate experiments are shown and are expressed as the mean ± SEM. ** $p < 0.01$, *** $p < 0.001$ vs. the control group.

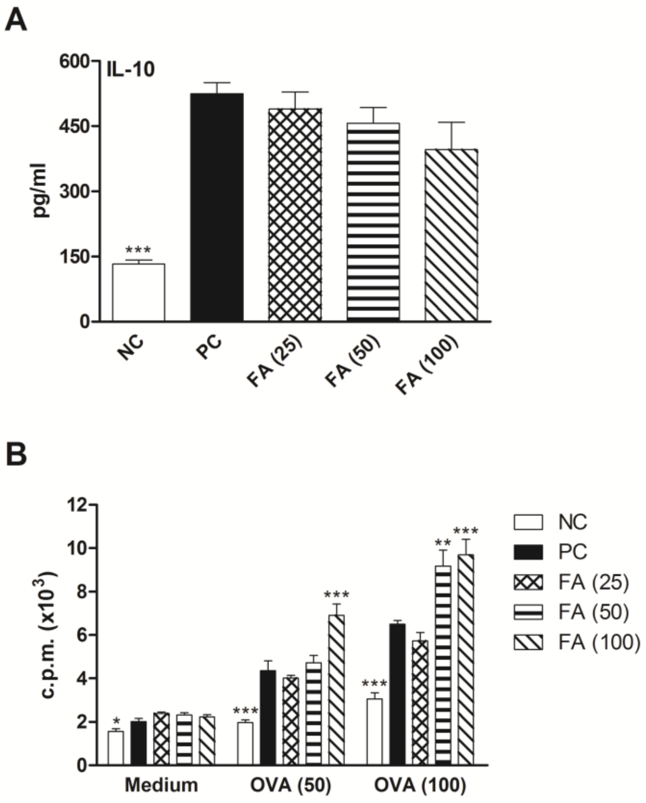

Figure S3: Treatment with ferulic acid enhanced the allergen-specific T-cell proliferative response in ovalbumin (OVA)-induced asthmatic mice. (A) Splenocytes (10^7 cells/ml) from FA-treated and control groups were stimulated with 50 μg/ml OVA in 24-well plates, and culture supernatants were collected after 72 h. Levels of IL-10 cytokine production was analyzed by an ELISA. Results are expressed as the mean ± SEM (n = 8 in each group). *** $p < 0.001$ vs. the PC group. (B) 3 x 10^5 spleen cells/well were stimulated with OVA (50 and 100 μg/ml) *in vitro*. After 5 days of culture, cell proliferation was estimated by [^{3}H]-thymidine incorporation assay. Results are expressed as the mean ± SEM (n = 8 in each group). * $p < 0.05$, *** $p < 0.001$ vs. the PC group.
